# Prognostic role of pretreatment blood lymphocyte count in patients with solid tumors: a systematic review and meta-analysis

**DOI:** 10.1186/s12935-020-1094-5

**Published:** 2020-01-10

**Authors:** Jiawen Zhao, Weijia Huang, Yongxian Wu, Yihuan Luo, Bo Wu, Jiwen Cheng, Junqiang Chen, Deyun Liu, Chengyang Li

**Affiliations:** 1 Department of Urology, The First Affiliated Hospital of Guangxi Medical University, 6 Shuangyong Road, Nanning, 530021 Guangxi Zhuang Autonomous Region China; 2 Department of Gastrointestinal Surgery, The First Affiliated Hospital of Guangxi Medical University, 6 Shuangyong Road, Nanning, 530021 Guangxi Zhuang Autonomous Region China

**Keywords:** Lymphocyte, Pretreatment, Prognosis, Solid tumor

## Abstract

**Background:**

To evaluate the prognostic value of pretreatment lymphocyte counts with respect to clinical outcomes in patients with solid tumors.

**Methods:**

Systematic literature search of electronic databases (Pubmed, Embase and Web of Science) up to May 1, 2018 was carried out by two independent reviewers. We included Eligible studies assessed the prognostic impact of pretreatment lymphocytes and had reported hazard ratios (HR) with 95% confidence intervals (CIs) for endpoints including overall survival (OS) and progression-free survival (PFS). Only English publications were included.

**Results:**

A total of 42 studies comprising 13,272 patients were included in this systematic review and meta-analysis. Low pretreatment lymphocyte count was associated with poor OS (HR = 1.27, 95% CI 1.16–1.39, *P* < 0.001, I^2^ = 58.5%) and PFS (HR = 1.27, 95% CI 1.15–1.40, *P* < 0.001, I^2^ = 25.7%). Subgroup analysis disaggregated by cancer type indicated that low pretreatment lymphocytes were most closely associated with poor OS in colorectal cancer followed by breast cancer and renal cancer.

**Conclusions:**

Low pretreatment lymphocyte count may represent an unfavorable prognostic factor for clinical outcomes in patients with solid tumors.

## Background

An increasing body of evidence suggests that immune status, an essential biological marker, is a key factor in carcinogenesis and cancer progression. Lymphocytes, such as those in the peripheral blood and tumor-infiltrating lymphocytes (TILs) constitute one of the most important effector mechanisms of anti-tumor immunity. Tumor cells are often surrounded by immune cells, especially lymphocytes. Tumor cells are distinguishable from healthy cells by the presence of tumor antigens which provide an immunological stimulus. Lymphocytes play an important role in anti-tumor immunity by inducing apoptosis and by suppressing the proliferation and migration of tumor cells [1–3]. High numbers of TILs were shown to be associated with inhibition of tumor progression and favorable prognosis in patients with hepatocellular carcinoma [4], colorectal cancers [5], and ovarian cancers [6]. Results of a meta-analysis suggest that TILs moderately influence the prognosis in diverse types of cancer; in particular, high number of intratumoral CD3+, CD4+ or CD8+ lymphocytes was associated with a lower risk of death and progression [2]. Numerous clinical studies have revealed that peripheral blood lymphopenia prior to initial treatment is associated with poor prognosis in various types of cancers, such as advanced carcinomas and sarcomas, cervical cancer, renal carcinoma, and bladder cancer [1, 7–9]. However, the inconsistent effect of pretreatment blood lymphocyte counts in patients with some publications cannot be ignored [10–15]. Moreover, the prognostic impact of lymphopenia in non-hematologic tumors has not been systematically analyzed. In order to reach a more reliable conclusion, a systematic review and meta-analysis to synthesize the evidence pertaining to pretreatment peripheral blood lymphocytes in patients with solid tumors is indispensable.

## Materials and methods

### Data sources and search strategy

The Preferred Reporting Items for Systematic Review and Meta-Analysis (PRISMA) were applied in the present study [16]. We conducted a systematic literature search in the PubMed, Web of Science, and Embase electronic databases to identify relevant studies published as of May 1, 2018. Combinations of the following keywords were used to retrieve articles: “lymphopenia”, “lymphocytosis”, “lymphocytes”, “tumor”, “carcinoma”, “cancer” and “prognosis” or “survival”.

### Study selection criteria

Studies that qualified the following criteria were included: (1) original articles published in English language; (2) studies that enrolled patients with pathologically confirmed solid tumors who had not received any treatment; (2) lymphocyte counts were measured prior to the first treatment (surgery and/or chemotherapy or radiotherapy or palliative therapy); (3) pretreatment lymphocytes were reported as a dichotomous variable; (4) assessed the prognostic impact of pretreatment lymphocytes and had reported hazard ratio (HR) with 95% confidence interval (CI); at least provided Kaplan–Meier survival curves from which HRs and 95% CIs could be calculated.

In case of duplicate publications based on the same dataset, only the article with the largest sample size was included. Letters, reviews, case-reports, expert opinions and conference abstracts were excluded from the present study.

Titles and abstracts of articles retrieved on initial search were independently screened by two investigators (W.H. and Y.L.) to eliminate irrelevant articles. Full texts of the remaining articles were reviewed against the above criteria to identify eligible studies. In case of any disagreement between the two reviewers, the final decision was made by a third reviewer (J.Z.).

### Data extraction and quality evaluation

Data pertaining to the following variables were independently extracted by two authors (W.H. and Y.L.): first author; publication year; region; study design; cancer type; sample size; disease stage; cut-off value; survival analysis; treatment details; and HR with corresponding 95% CI for OS and/or PFS. Survival outcomes obtained on multivariate analysis were accorded precedence over those obtained on univariate analysis.

Two investigators (W.H. and Y.L.) independently assessed the quality of each study according to the Newcastle–Ottawa Scale (NOS); any disagreement was resolved by consensus [17]. Newcastle–Ottawa Scale mainly includes selection, comparability, and evaluation of outcomes. On a scale of 0 to 9, a study with score of ≥ 6 was considered as a high-quality study. However, quality assessment was not an exclusion criterion for eligible studies.

### Statistical analysis

We extracted the HRs and 95% CIs of the ratio for low pretreatment lymphocytes over high pretreatment lymphocytes from each eligible study for OS and/or PFS. The endpoints of survival were OS and/or PFS mainly because the two endpoints were frequently used in the included studies. Meta-analysis was performed to evaluate the prognostic effect of pretreatment lymphocytes in patients with solid tumors for each of the endpoints (OS/PFS). Extracted data were pooled using the Stata 12.0 (STATA Corporation, College Station, TX, USA). Cochrane Q test and the *I*^*2*^ statistic were used to test the heterogeneity among the studies included in the pooled analysis. In the absence of significant heterogeneity (*P* > 0.1 and *I*^*2*^ < 50%), the fixed effects model was used for pooled analysis [18]; otherwise, the random-effects model was used. Pooled HR > 1 was considered indicative of worse survival outcome of patients with low baseline lymphocytes. If the 95% CI did not overlap 1, the result was considered statistically significant. Subgroup analyses were performed to investigate the association of pretreatment lymphocyte counts with variables such as region, cancer type, disease stage, cut-off value, survival outcomes, and treatment scheme. Moreover, sensitivity analyses were performed by sequential elimination of one study at a time to explore its potential impact on the heterogeneity. We further used funnel plots and Egger’s test to examine the influence of publication bias on the pooled OS and PFS, respectively. All statistical tests were two-sided and *P* < 0.05 indicated statistical significance.

## Results

### Search and selection of studies

**Fig. 1:**
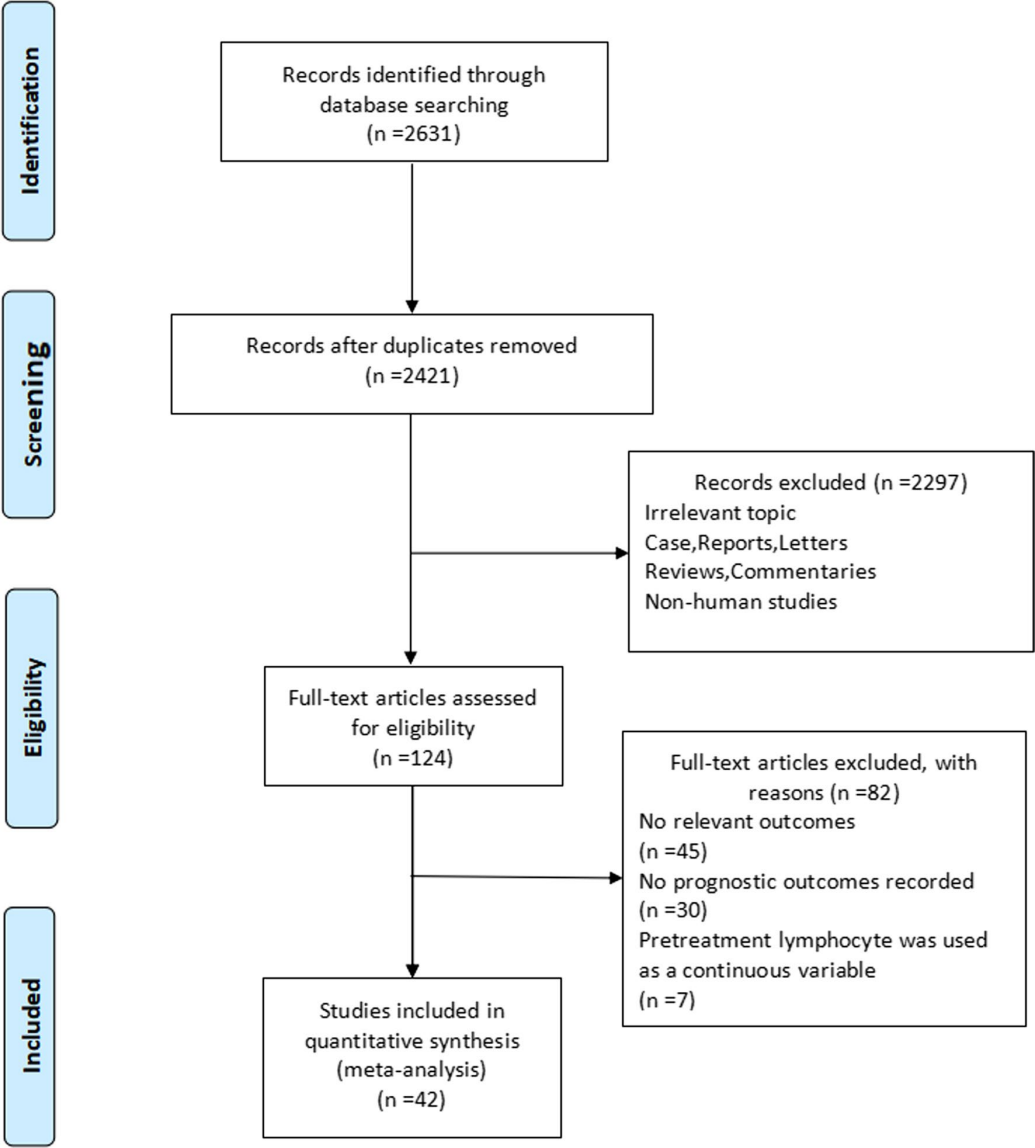
Schematic illustration of the meta-analysis

As illustrated in Fig. 1, a total of 2631 articles were retrieved on initial database search. Of these, 2507 articles were removed as irrelevant and duplicate articles. After full-text review, 75 were excluded due to lack of available information. Seven studies that reported lymphocytes count as a continuous variable were excluded. Finally, a total of 42 studies with a combined study population of 13,272 patients were considered eligible for inclusion [1, 7–15, 19–50]. The articles were published in the period from 2005 to 2018. The most common types of cancers in the included studies were lung cancer (n = 5), followed by nasopharyngeal cancer (n = 4) and renal cancer (n = 4). All the included studies had collected data retrospectively. Characteristics of included articles are described in Table 1.

**Table 1:** Main characteristics of the included studies in the meta-analysis

First author	Year	Region	Disease site	Disease stage	Inclusion period	No. of patients	Age	Treatment	Analysis of survival	Cut off value (× 10^9^/L)	Study design	Follow-up (months)	Outcome reported	NOS score
Yang [19]	2018	China^A^	Hypopharyngeal cancer	Non metastatic	2009–2014	197	NR	Chemo + Resection	Univariate	1.7	Retrospective	30.95^b^	OS	8
Pang [20]	2018	China^A^	Hepatocellular cancer	Non metastatic	2002–2016	470	52.2^a^	Resection	Univariate	0.7	Retrospective	29^b^	OS	7
Margetts [21]	2018	China^A^	Hepatocellular cancer	Mixed	2007–2013	585	60^b^	Resection + Chemo	Multivariate	1.3	Retrospective	NR	OS	7
Liu [22]	2018	China^A^	Nasopharyngeal cancer	Mixed	2007–2012	413	45^b^	Chemo	Univariate	1.315	Retrospective	NR	OS, PFS	6
Zhao^c^ [14]	2017	China^A^	Advanced cancer	Mixed	2013–2015	378	64^b^	Palliative therapy	Multivariate	0.8	Retrospective	14.83^b^	OS	6
Zhao^c^ [14]	2017	China^A^	Advanced cancer	Mixed	2013–2015	378	64^b^	Palliative therapy	Multivariate	1.1	Retrospective	14.83^b^	OS	6
Zhao^c^ [14]	2017	China^A^	Advanced cancer	Mixed	2013–2015	378	64^b^	Palliative therapy	Multivariate	1.5	Retrospective	14.83^b^	OS	6
Zhao [14]	2017	China^A^	Advanced cancer	Mixed	2013–2015	106	64^b^	Palliative therapy	Multivariate	0.8	Retrospective	16.97^b^	OS	6
He [15]	2017	China^A^	Hepatocellular cancer	Non metastatic	2007–2015	216	53^b^	Chemo	Univariate	0.8	Retrospective	NR	OS	8
Bobdey [23]	2017	India^A^	Oral cancer	Mixed	2007–2008	471	50^a^	Chemo	Univariate	1.98	Retrospective	22^b^	OS	6
Xu [24]	2017	China^A^	Glioblastoma	Non metastatic	2010–2015	166	50^b^	Resection	Multivariate	1.9	Retrospective	14^b^	OS	7
Zhang [25]	2017	China^A^	Gallbladder cancer	Mixed	2001–2013	98	63^a^	Resection	Univariate	2.06	Retrospective	NR	OS	8
Sorensen [26]	2017	Denmark^NA^	MBDex	Metastatic	2003–2013	270	64^b^	Resection	Multivariate	1.37	Retrospective	8.82^b^	OS	6
Oh [27]	2017	Korea^A^	Colorectal cancer	Mixed	2006–2011	261	65^b^	Resection	Univariate	1.83	Retrospective	78^b^	OS	7
Wu [7]	2016	America^NA^	Cervical cancer	Non metastatic	1998–2013	71	49^a^	Chemo	Multivariate	1.0	Retrospective	30.4^b^	OS, PFS	8
Sun [28]	2016	China^A^	Gastric cancer	Non metastatic	2000–2012	873	59^b^	Resection	Univariate	3	Retrospective	36^b^	OS, PFS	8
Sun [29]	2016	China^A^	Nasopharyngeal cancer	Non metastatic	2008–2011	251	46^b^	Chemo	Multivariate	1.5	Retrospective	41^b^	OS,PFS	7
Kou [30]	2016	China^A^	Esophagus cancer	Metastatic	2005–2013	215	58^b^	Chemo	Multivariate	1.0	Retrospective	120	OS	6
Joseph [9]	2016	UK^NA^	Bladder cancer	Non metastatic	2009–2014	131	68^b^	Chemo	Multivariate	1.5	Retrospective	17^b^	OS	8
Eo [31]	2016	Korea^A^	Endometrial cancer	Non metastatic	2005–2014	255	44^b^	Resection	Univariate	1.526	Retrospective	51.3^b^	OS	7
d’Engremont [32]	2016	France^NA^	Pancreatic cancer	Non metastatic	2000–2010	390	NR	Resection	Multivariate	1.0	Retrospective	66.6^b^	OS	6
Deng [33]	2016	China^A^	Gallbladder cancer	Mixed	2002–2012	315	NR	Resection	Multivariate	1.5	Retrospective	9^b^	OS	6
Cho [34]	2016	Korea^A^	Lung cancer	Non metastatic	2001–2014	73	65^a^	Radiotherapy	Univariate	1.747	Retrospective	22^b^	OS, PFS	7
Cho [35]	2016	Korea^A^	Cervical cancer	Mixed	2001–2012	124	57^b^	Chemoradiotherapy	Multivariate	1.5	Retrospective	63^b^	PFS	6
Berardi [36]	2016	Italy^NA^	Lung cancer	Mixed	2009–2014	401	68^a^	Chemo	Univariate	1.5	Retrospective	80	OS, PFS	7
Zhou [50]	2016	China^A^	Gastric cancer	Non metastatic	2006–2008	451	NR	Resection	Univariate	1.5	Retrospective	37.7^b^	OS	6
Wild [10]	2015	America^NA^	Pancreatic cancer	Non metastatic	1997–2011	101	62^b^	Chemo	Univariate	1.0	Retrospective	10.1^b^	OS	6
Santoni [11]	2015	Italy^NA^	Renal cancer	Mixed	2005–2014	151	64^a^	Chemo	Univariate	1.5	Retrospective	51.6^b^	OS, PFS	7
Rochet [12]	2015	America^NA^	Stage III Melanoma	Non metastatic	2000–2010	153	59^b^	Resection	Multivariate	2.1	Retrospective	30^b^	OS	7
Rochet [12]	2015	America^NA^	Stage IV Melanoma	Metastatic	2000–2010	74	56^b^	Resection	Multivariate	1.9	Retrospective	24^b^	OS	7
Mehrazin [37]	2015	America^NA^	Renal cancer	Non metastatic	2000–2013	192	62^a^	Resection	Multivariate	1.3	Retrospective	38.7^b^	OS	6
Ku [38]	2015	UK^NA^	Urothelial cancer	Non metastatic	1999–2011	419	65.1^b^	Resection	Multivariate	1.0	Retrospective	37.7^b^	OS	7
Jin [39]	2014	China^A^	Nasopharyngeal cancer	Metastatic	2006–2011	229	45^b^	Chemo	Multivariate	1.0	Retrospective	84	OS	7
Paik^c^ [13]	2014	Korea^A^	Colorectal cancer	Non metastatic	2006–2009	600	62.3^a^	Resection	Univariate	1.0	Retrospective	27,4^b^	OS	8
Paik^c^ [13]	2014	Korea^A^	Colorectal cancer	Non metastatic	2006–2009	600	62.3^a^	Resection	Univariate	3.0	Retrospective	27,4^b^	OS	8
Kumagai [40]	2014	Japan^A^	Lung cancer	Non metastatic	2007–2012	302	67^b^	Resection	Multivariate	1.4	Retrospective	33.4^b^	OS	7
DeGiorgi [41]	2014	Italy^NA^	Renal cancer	Metastatic	2006–2011	181	NR	Chemo	Multivariate	1.0	Retrospective	NR	OS, PFS	7
Zhang [42]	2013	China^A^	Lung cancer	Mixed	1999–2006	142	57.5^a^	Resection	Multivariate	1.8	Retrospective	NR	OS	7
Saroha [8]	2013	America^NA^	Renal cancer	Non metastatic	1994–2008	430	60.2^a^	Resection	Multivariate	1.3	Retrospective	33.5^b^	OS	6
Manuel [43]	2012	France^NA^	Breast cancer	Metastatic	NR	66	NR	Chemo	Univariate	1.0	Retrospective	18.8^b^	OS	8
Manuel [43]	2012	France^NA^	Pancreatic cancer	Metastatic	NR	67	NR	Chemo	Univariate	1.0	Retrospective	14.3^b^	OS	8
He^c^ [44]	2012	China^A^	Nasopharyngeal cancer	Non metastatic	2005–2007	1410	46.1^a^	Chemo	Multivariate	1.69	Retrospective	41^b^	OS, PFS	7
He^c^ [44]	2012	China^A^	Nasopharyngeal cancer	Non metastatic	2005–2007	1410	46.1^a^	Chemo	Multivariate	2.06	Retrospective	41^b^	OS, PFS	7
He^c^ [44]	2012	China^A^	Nasopharyngeal cancer	Non metastatic	2005–2007	1410	46.1^a^	Chemo	Multivariate	2.53	Retrospective	41^b^	OS, PFS	6
DeGiorgi [45]	2012	America^NA^	Breast cancer	Metastatic	2004–2008	195	54^b^	Chemo	Multivariate	1.0	Retrospective	NR	OS, PFS	7
Ceze [46]	2011	France^NA^	Colorectal cancer	Non metastatic	1999–2007	260	64.8^a^	Chemo	Multivariate	1.0	Retrospective	15^b^	OS, PFS	6
Teramukai^c^ [47]	2009	Japan^A^	Lung cancer	Mixed	2001–2005	388	65^b^	Chemo	Multivariate	1.082	Retrospective	18.9^b^	OS, PFS	7
Teramukai^c^ [47]	2009	Japan^A^	Lung cancer	Mixed	2001–2005	388	65^b^	Chemo	Multivariate	1.386	Retrospective	18.9^b^	OS, PFS	7
Teramukai^c^ [47]	2009	Japan^A^	Lung cancer	Mixed	2001–2005	388	65^b^	Chemo	Multivariate	1.821	Retrospective	18.9^b^	OS, PFS	7
Ray-Coquard [1]	2009	France^NA^	Breast cancer	Metastatic	NR	287	NR	Chemo	Multivariate	1.0	Retrospective	138	OS, PFS	8
Ray-Coquard [1]	2009	France^NA^	Soft tissue sarcoma	Metastatic	NR	193	NR	Chemo	Multivariate	1.0	Retrospective	90	OS, PFS	8
LeScodan [48]	2007	France^NA^	Brain metastases	Metastatic	1998–2003	132	54.9^b^	Chemo	Multivariate	0.7	Retrospective	25^b^	OS	7
Claude [49]	2005	France^NA^	Brain metastases	Metastatic	1991–2001	120	54^b^	Radiotherapy	Multivariate	0.7	Retrospective	67^b^	OS	7

*NR* not report, *OS* overall survival, *PFS* progression free survival, *MBDex* metastatic bone disease in the extremities

^a^Mean; ^b^median; ^c^The same patients sources in different cut-off values; ^A^Asian; ^NA^Non-Asian

### Relationship between pretreatment lymphocytes and survival outcomes

#### Overall survival

**Fig. 2:**
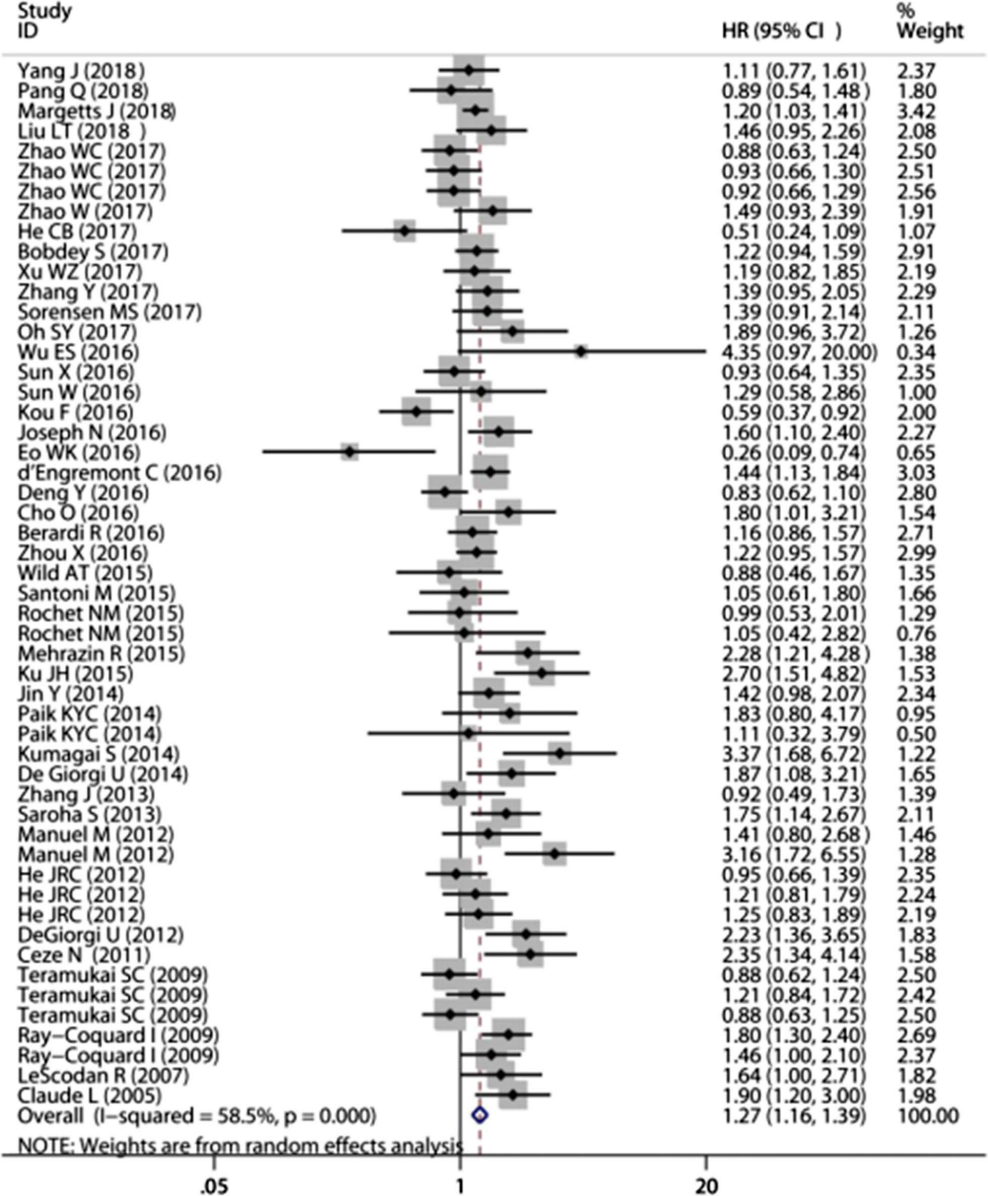
Forest plots for the association between pretreatment lymphocyte and overall survival

A total of 41 studies involving 45 cohorts (13,148 patients) investigated the association between pretreatment lymphocytes and OS. The median cut-off value of pretreatment lymphocytes in the included cohorts was 1.3425 (range: 0.7–3.0). In 16 articles, the HRs and 95% CIs were obtained on univariate analysis, while 25 articles had calculated HR on multivariate analysis. Overall, low pretreatment lymphocyte counts were associated with poor OS (HR = 1.27, 95% CI 1.16–1.39, *P* < 0.001) (Fig. 2). There was moderate heterogeneity among studies and thus a random-effects model was used (*I*^*2*^ = 58.5%). Subgroup analysis stratified by main clinical features (tumor type, cut-off value, survival analysis, and treatment) was performed. On subgroup analysis stratified by cancer type, low pretreatment lymphocytes were most closely associated with poor OS in colorectal cancer (n = 3, HR = 1.96, 95% CI 1.36–2.83, *P* < 0.001, *I*^*2*^ = 0), followed by breast cancer (n = 3, HR = 1.82, 95% CI 1.43–2.31, *P* < 0.001, *I*^*2*^ = 0), and renal cancer (n = 4, HR = 1.65, 95% CI 1.22–2.24, *P* = 0.001, *I*^*2*^ = 24.3%) (Table 2). On subgroup analysis stratified by pretreatment lymphocytes cut-off value, the largest effect size was observed in the cut-off value ≤ 1.0 subgroup (n = 17, HR = 1.46; 95% CI 1.21–1.77, *P* < 0.001, *I*^*2*^ = 67.6%); followed by the 1.0 ˂ cut-off ≤ 2.0 subgroup (n = 23, HR = 1.18; 95% CI 1.06–1.31, *P* = 0.004, *I*^*2*^ = 49.6%). Cut-off ˃ 2.0 subgroup was not associated with poor OS (n = 5, HR = 1.16; 95% CI 0.96–1.39, *P* = 0.121, *I*^*2*^ = 0). On subgroup analysis stratified by disease stage, both non-metastatic (n = 21, HR = 1.32, 95% CI 1.12–1.54, *P* ˂ 0.001, *I*^*2*^= 58.0%) and metastatic subgroups (n = 10, HR = 1.54, 95% CI 1.24–1.92, *P* ˂ 0.001, *I*^*2*^= 60.2%) were significantly associated with unfavorable OS. However, for the mixed subgroup (patients with both non-metastatic and metastatic disease), the pooled HR was 1.09 (n = 11, HR = 1.09, 95% CI 0.98–1.20, *P* = 0.107, *I*^*2*^ = 26.2%). No significant differences in survival outcomes were observed on subgroup analysis stratified by treatment or by type of survival analysis (univariate analysis vs. multivariate analysis). Further, sensitivity analysis showed that the pooled HRs for OS were not significantly affected by elimination of any individual study from the pooled analysis. The funnel plot was roughly symmetrical and Egger’s test showed no significant effect of publication bias on the results of the meta-analysis (P = 0.188 for OS).

**Table 2:** Subgroup analysis of the meta-analysis for OS

Subgroup	No. of studies	No. of patients	Pooled HR	95% CI	*P*	Heterogeneity test		Statistical method
						*I*^*2*^	*P*	
Treatment								
Resection [8, 12, 13, 20, 24–28, 31–33, 37, 38, 40, 42, 50]	17	5861	1.30	1.08–1.55	0.004	61.5%	<0.001	Random
Chemo [1, 7, 9–11, 15, 22, 23, 29, 30, 36, 39, 41, 43–47]	18	5687	1.64	1.00–2.71	< 0.001	60.0%	<0.001	Random
Analysis of survival								
Multivariate [1, 7–9, 12, 14, 21, 24, 26, 29, 30, 32, 33, 37–42, 44–49]	25	7612	1.31	1.16–1.47	< 0.001	63.6%	<0.001	Random
Univariate [10, 11, 13, 15, 19, 20, 22, 23, 25, 27, 28, 31, 34, 36, 43, 50]	16	5536	1.20	1.02–1.40	0.023	46.6%	0.016	Random
Cut-off value								
≤ 1.0 [1, 7, 10, 13–15, 20, 30, 32, 38, 39, 41, 43, 45, 46, 48, 49]	17	4437	1.46	1.21–1.77	< 0.001	67.6%	<0.001	Random
1.0 to < 2.0 [8, 9, 11, 12, 14, 19, 21–24, 26, 27, 29, 31, 33, 34, 36, 40, 42, 44, 47, 50]	22	7646	1.18	1.06–1.31	0.004	49.6%	0.002	Random
≥ 2.0 [12, 13, 25, 28, 44]	5	4544	1.16	0.96–1.39	0.121	0.0%	0.760	Random
Disease site								
Colorectal cancer [13, 27, 46]	3	1121	1.96	1.36–2.83	< 0.001	0.0%	0.737	Random
Breast cancer [1, 43, 45]	3	454	1.82	1.43–2.31	< 0.001	0.0%	0.509	Random
Renal cancer [8, 11, 37, 41]	4	954	1.65	1.22–2.24	0.001	24.3%	*0.265*	Random
Lung cancer [34, 36, 40, 42, 47]	5	1306	1.20	0.92–1.57	0.177	63.9%	0.011	Random
Pancreatic cancer [10, 32, 43]	3	558	1.56	0.88–2.15	0.129	73.5%	0.023	Random
Nasopharyngeal cancer [22, 29, 39, 44]	4	2303	1.23	1.03–1.46	0.017	0.0%	0.701	Random
Gallbladder cancer [25, 33]	2	511	1.05	0.637–1.75	0.828	77.7%	0.034	Random
Gastric cancer [28, 50]	2	1324	1.10	0.85–1.43	0.442	29.9%	0.232	Random
Disease stage								
Non metastatic [7–10, 12, 13, 15, 19, 20, 24, 28, 29, 31, 32, 34, 37, 38, 40, 44, 46, 50]	21	7437	1.32	1.12–1.54	< 0.001	58.0%	0.001	Random
Metastatic [1, 12, 26, 30, 39, 41, 43, 45, 48, 49]	10	2108	1.54	1.24–1.92	< 0.001	60.2%	0.004	Random
Mixed [11, 14, 21–23, 25, 27, 33, 36, 42, 47]	11	3603	1.09	0.98–1.20	0.107	26.2%	0.160	Random
Region								
Asian [13–15, 19–25, 27–31, 33, 34, 39, 40, 42, 44, 47, 50] (China, India, Korea, Japan)	23	8422	1.10	0.99–1.21	0.08	48.6%	0.001	Random
Non-Asian [1, 7–12, 26, 32, 36–38, 41, 43, 45, 46, 48, 49] (Denmark, America, UK, France, Italy)	18	4726	1.27	1.16–1.39	< 0.001	32.0%	0.080	Random

#### Progression-free survival

**Fig. 3:**
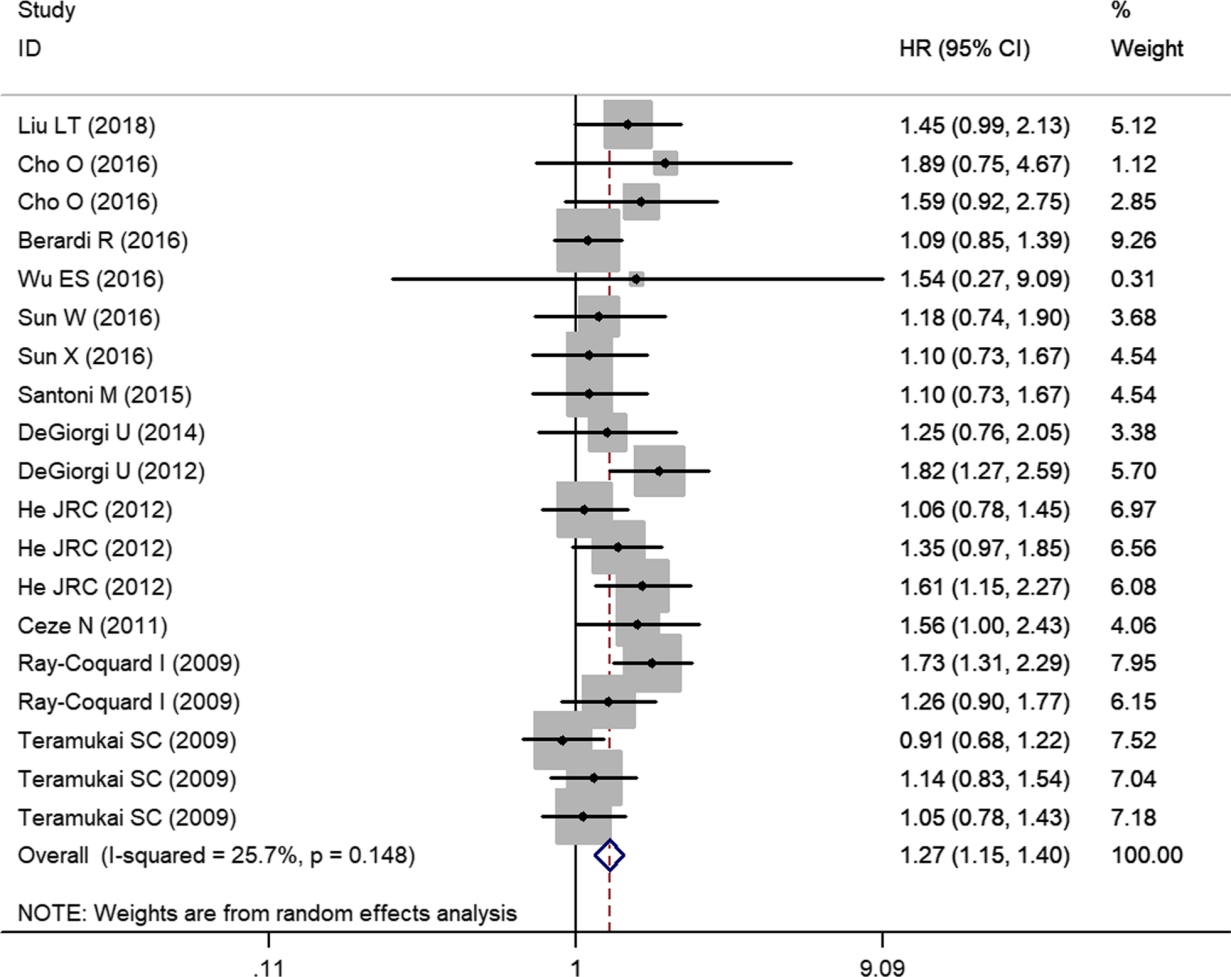
Forest plots for the association between pretreatment lymphocyte and progression-free survival

A total of 14 studies comprising of 18 cohorts (5147 patients) were included in the analysis of HRs for PFS. The median cut-off value for pretreatment lymphocytes was 1.50 (range: 1–3). In 9 articles, the HRs and 95% CIs were obtained by multivariable analysis; while 5 articles had calculated HRs and 95% CIs by univariate analysis. Overall, low pretreatment lymphocyte counts were significantly associated with worse PFS (Fig. 3). Owing to the lack of significant heterogeneity (*I*^*2*^ = 25.7%), the fixed-effects model was used for pooled analysis. On subgroup analysis stratified by cancer type, low pretreatment lymphocytes was most closely associated with poor PFS in patients with breast cancer (n = 2, HR = 1.76, 95% CI 1.42–2.20, *P* ˂ 0.001, *I*^*2*^ = 0) (Table 3). Likewise, the funnel plot was roughly symmetrical and Egger’s test revealed no significant influence of publication bias (*P *= 0.267 for PFS).

**Table 3:** Subgroup analysis of the meta-analysis for PFS

Subgroup	No. of studies	No. of patients	Pooled HR	95% CI	*P*	Heterogeneity test		Statistical method
						*I*^*2*^	*P*	
Analysis of survival								
Multivariate	9 [1, 7, 29, 35, 41, 44–47]	2487	1.30	1.14–1.47	< 0.001	37.1%	0.080	Fixed
Univariate	5 [11, 22, 28, 34, 36]	2660	1.19	1.01–1.40	0.036	0.0%	0.441	Fixed
Cut-off value								
≤ 1.0	5 [1, 7, 41, 45, 46]	1187	1.55	1.32–1.82	< 0.001	0.0%	0.617	Fixed
> 1.0	9 [11, 22, 28, 29, 34–36, 44, 47]	3960	1.11	0.99–1.24	0.053	0.0%	0.643	Fixed
Disease site								
Nasopharyngeal cancer	3 [22, 29, 44]	2074	1.31	1.12–1.53	0.001	0.0%	0.444	Fixed
Breast cancer	2 [1, 45]	482	1.76	1.42–2.20	< 0.001	0.0%	0.820	Fixed
Renal cancer	2 [11, 41]	332	1.15	0.84–1.59	0.36	0.0%	0.690	Fixed
Disease stage								
Non metastatic	6 [7, 28, 29, 34, 44, 46]	2814	1.34	1.14–1.56	< 0.001	0.0%	0.612	Fixed
Metastatic	3 [1, 41, 45]	856	1.54	1.30–1.84	< 0.001	15.2%	0.316	Fixed
Mixed	5 [11, 22, 35, 36, 47]	1477	1.10	0.97–1.24	0.138	0.0%	0.528	Fixed
Region								
Asian(China, Korea, Japan)	7 [22, 28, 29, 34, 35, 44, 47]	3408	1.20	1.07–1.34	0.002	20.2%	0.257	Fixed
Non Asian(America, France, Italy)	7 [1, 7, 11, 36, 41, 45, 46]	1739	1.37	1.20–1.55	< 0.001	31.6%	0.176	Fixed

## Discussion

To the best of our knowledge, this is the first systematic review and meta-analysis that comprehensively summarizes the association between lymphocyte count and cancer survival. Current meta-analysis included a total of 42 studies with a combined study population of 13,272 patients and provides evidence that low lymphocyte counts are associated with shorter OS and PFS in patients with non-hematologic tumors. There was moderate heterogeneity among studies in the analysis of OS (*I*^*2*^ = 58.5%) but not that of PFS (*I*^*2*^ = 25.7%). Subsequently, on subgroup analysis by tumor location, the highest effect size with respect to OS was observed in patients with colorectal cancer followed by those with breast cancer and renal cancer. Intriguingly, we found a significant reduction in heterogeneity in subgroups of patients with colorectal cancer (*I*^*2*^ = 0), breast cancer (*I*^*2*^ = 0) and renal cancer (*I*^*2*^ = 24.3%) although moderate heterogeneity was observed (*I*^*2*^ = 58.5%) in the pooled analysis. Moreover, when stratified by disease stage in the analysis of OS and PFS, low lymphocyte count was an adverse prognostic factor in both non-metastatic and metastatic subgroups. This suggests that lymphocytes are involved in several stages of cancer development. Moreover, the negative prognostic effect on OS and PFS was consistent in subgroups stratified by cut-off value and type of survival analysis.

Patients with pretreatment lymphopenia have significantly worse survival than those of patients with normal lymphocyte counts in the context of several malignancies [1, 7–9]. Lymphocytes are known to play a role in cellular and humoral anti-tumor immune responses. Activated and proliferating lymphocytes play a role in cytotoxic cell death and inhibit tumor cell proliferation and migration. Chew et al. observed lymphocyte recruitment and proliferation in tumor areas devoid of tumor cell proliferation and rich in tumor cell apoptosis [4]. Therefore, lymphopenia may reflect poor host immunity against cancer and a favorable microenvironment for tumor growth. The underlying mechanism of pretreatment lymphopenia in solid tumors has not been fully clarified and is probably multifactorial. It is widely believed that lymphopenia may result from increased lymphocyte apoptosis and/or altered lymphocyte homeostasis. Kim et al. demonstrated that increased expression of Fas ligand (FasL) in tumor cells mediated apoptosis of TILs as well as circulating lymphocytes, which conferred immune privilege to tumors [51]. Increased numbers of apoptotic peripheral T lymphocytes have been detected in patients with gastric cancer [52]. Over-production of immunosuppressive cytokines such as transforming growth factor (TGF-β) and IL-10 by tumor cells specially during tumor growth may suppress different effector pathways of the immune response [53, 54]. Exposure to TGF-β reduced the expressions of apoptotic activators (such as perforin and granzyme A and B) on cytotoxic T cells that infiltrated the tumor tissues. Additionally, tumor growth increases the recruitment of CD4+ regulatory T cells that secrete IL-10 and TGF-β and suppress effector CD8+ T cell responses [55]. IL-10 exerts an inhibitory effect on major histocompatibility complex (MHC) class I antigen presentation. Dummer et al. observed excessive expression of immunosuppressive factor IL-I0 in metastatic lesions and in cultured cells from metastases; they inferred that this cytokine plays a key role in tumor progression [56]. Although numerous studies previously focused on T-cell-mediated immunity, B cells play an equally prominent role in modulating anti-tumor immune responses and in carcinogenesis. B cells are classically known for their role as producers of antibodies. Tumor-infiltrating B cells have relation to improved survival in cervical cancer and non-small cell lung cancer [57, 58]. Results from these clinical observations suggest that the potential mechanisms underlying B-cell anti-tumor immunity may involve tumor-infiltrating B cells could recruit and retain T cells at the tumor site, thus facilitating and sustaining T-cell responses that inhibit tumor development. Moreover, tumor-infiltrating B cells may function as antigen-presenting cells to aid in anti-tumor immunity [57, 59]. Thus, it may be possible to generate more amplified and prolonged immune responses at the tumor site by promoting cooperative interactions of B cells and T cells. Collectively, these findings suggest that lymphopenia may be a result of cancer-induced immune suppression that drives tumor progression.

Neutrophil–lymphocyte ratio (NLR) has been identified as an independent prognostic factor in many solid tumors; a high NLR ratio was shown to be associated with inferior outcomes [60–62]. Nevertheless, it includes two potentially independent biological factors; high NLR indicates an increase in neutrophil and/or decreased total lymphocyte count. A meta-analysis of one hundred studies (combined n = 40,559) conducted by Templeton et al. revealed that high NLR is associated with adverse OS, CSS, PFS, or DFS in many solid tumors [63]. The prognostic impact of NLR may be explained by the association of high NLR with inflammation. However, at the same time, the authors admitted that the confounding effect of concurrent inflammatory conditions cannot be completely excluded because high NLR has also been shown to be of prognostic relevance in non-cancerous conditions such as acute pancreatitis [64] and cardiac events [65]. Joseph suggested that the prognostic value of high neutrophil–lymphocyte ratio may actually be driven by lymphocytopenia rather than neutrophilia in patients with bladder cancer [9]. Similar results have been reported elsewhere; lymphocyte count was shown to exert a stronger impact on the neutrophil-to-lymphocyte ratio in clear cell renal carcinoma and pancreatic cancer [8, 32]. Therefore, based on these observations, we evaluated the prognostic value of pretreatment peripheral blood lymphocyte counts with respect to clinical outcomes in patients with solid tumors.

Lymphocytopenia is not just a parameter related to cancer survival but may also reflect a biological mechanism that promotes tumor progression. Of note, adjunctive treatment for reversal of lymphopenia or to increase lymphocyte counts has also been proposed by some authors. Restoration of lymphocyte homeostasis may lead to activation of effector cytotoxic and helper T cells and result in a more potent antitumor immune response. IL-2 was used for treatment of patients with metastatic melanoma. Recombinant human IL-7 (rhIL-7) was shown to improve the immune function of patients with lymphopenia by promoting peripheral T cell expansion and suppressing the immunosuppressive network [66].

In view of the possible impact of different cut-off values of pretreatment lymphocytes on prognosis, we observed the largest effect size in the cut-off ≤ 1.0 subgroup; the next was the 1.0 < cut-off ≤ 2.0 subgroup. Nonetheless, the cut-off > 2.0 subgroup was not associated with poor OS. Similar results were obtained on subgroup analysis of PFS. Hence, a relatively lower pretreatment lymphocytes cut-off value may have a better discriminative prognostic value. However, optimal pretreatment lymphocytes cutoff value for various types of cancers needs further research.

Undoubtedly, our research has several limitations. First, our meta-analysis was based on HR and 95% CIs extracted from retrospective studies. Due to the inherent limitations of retrospective studies including heterogeneity with respect to data selection and analysis, our pooled data might be susceptible to biases and may be biased towards positive results. Second, moderate heterogeneity was observed in the analysis of OS and the sources of this heterogeneity remain unclear; however, no significant heterogeneity was observed in the analysis of PFS. This is likely attributable to inclusion of more than 40 cohorts comprising of 13,000 patients with different tumors and from various countries. As yet, we have not found any meta-analysis that determined the prognostic value of pretreatment lymphocytes in any malignancy. Our goal was to gain a comprehensive understanding of the prognostic value of lymphocytes in patients with solid tumors. Therefore, the moderate heterogeneity observed in the analysis of OS is reasonably expected. Third, in 16 out of the 42 studies, the HRs were calculated on univariate analysis. Compared with data from multivariate analysis, HR and 95% CI calculated on univariate analysis is more likely to lead to an overestimation of the prognostic value. Therefore, we conducted subgroup analysis of univariate analysis and multivariate analysis and the statistical significance was stable; moreover, the multivariate analysis subgroup even had a larger effect size.

## Conclusion

Peripheral blood lymphocytes is a simple and routine index in clinical work. To the best our knowledge, we have not found any meta-analysis that determined the prognostic value of pretreatment lymphocytes in any malignancy. Our meta-analysis provides evidence that pretreatment lymphocyte might be a potential biomarker for survival in patients with solid tumors. However, the present meta-analysis was based on observational studies; we could not demonstrate a cause-effect relationship between pretreatment lymphocyte and survival in patients with solid tumors. Further prospective large-scale investigations are required to explore whether reversing lymphopenia can be a new target for cancer treatment and to increase the understanding of its role in disease pathogenesis.

## Data Availability

The datasets analyzed during the current study are available from the corresponding author on reasonable request.

## Abbreviations

HR: hazard ratio
CL: confidence interval
OS: overall survival
PFS: progression-free survival
NOS: Newcastle–Ottawa Scale
TILs: tumour-infiltrating lymphocytes
PRISMA: Preferred Reporting Items for Systematic Review and Meta-Analysis
FasL: Fas ligand
TGF-β: transforming growth factor
MHC: major histocompatibility complex
NLR: neutrophil–lymphocyte ratio
